# Brain circuits underlying visual stability across eye movements—converging evidence for a neuro-computational model of area LIP

**DOI:** 10.3389/fncom.2014.00025

**Published:** 2014-03-11

**Authors:** Arnold Ziesche, Fred H. Hamker

**Affiliations:** ^1^Artificial Intelligence, Computer Science, Chemnitz University of TechnologyChemnitz, Germany; ^2^Otto Creutzfeldt Center for Cognitive and Behavioral Neuroscience, University of MuensterMuenster, Germany

**Keywords:** space perception, computational model, predictive remapping, eye position signal, corollary discharge, saccadic suppression of displacement

## Abstract

The understanding of the subjective experience of a visually stable world despite the occurrence of an observer's eye movements has been the focus of extensive research for over 20 years. These studies have revealed fundamental mechanisms such as anticipatory receptive field (RF) shifts and the saccadic suppression of stimulus displacements, yet there currently exists no single explanatory framework for these observations. We show that a previously presented neuro-computational model of peri-saccadic mislocalization accounts for the phenomenon of predictive remapping and for the observation of saccadic suppression of displacement (SSD). This converging evidence allows us to identify the potential ingredients of perceptual stability that generalize beyond different data sets in a formal physiology-based model. In particular we propose that predictive remapping stabilizes the visual world across saccades by introducing a feedback loop and, as an emergent result, small displacements of stimuli are not noticed by the visual system. The model provides a link from neural dynamics, to neural mechanism and finally to behavior, and thus offers a testable comprehensive framework of visual stability.

## 1. Introduction

When we shift our gaze, which occurs about three times per second, the retinal image changes, yet we perceive the environment as a stable entity. Well-established experimental findings suggest that the maintenance of a stable world percept is an active, constructive process which relies on corollary discharge signals from the motor system (Sperry, 1950; Von Holst and Mittelstaedt, 1950; Sommer and Wurtz, 2006; Klier and Angelaki, 2008; Melcher and Colby, 2008; Wurtz, 2008; Medendorp, 2011). Thus, our percept of space is not only determined by the images from the eyes. The general idea is that a corollary of a motor plan can inform brain areas involved in perception about the upcoming motor action allowing for compensatory or predictive computations. In addition, predictive remapping, first observed in the lateral intraparietal area (LIP) (Duhamel et al., 1992; Kusunoki and Goldberg, 2003), has often been tied to the maintenance of visual stability across eye movements. Predictive remapping refers to the observation that some neurons with retinotopic receptive fields, i.e., fields that move with the eyes, become responsive to a stimulus placed in the future receptive field (FRF) of the cell prior to saccade or at least respond with a shorter latency compared to a condition without saccade. The FRF denotes the receptive field (RF) of the cell after the eye movement. Thus, just before saccade onset these neurons process a stimulus that will be in their RF after saccade. However, neither the exact mechanism of predictive remapping nor its function in the perception of a stable world is understood. On the contrary, Bays and Husain (2007) casted doubt on the role of predictive remapping for visual stability and instead proposed that remapping served a non-perceptual role, such as action control.

Other than corollary discharge, stimulus localization across saccades could involve eye position information (Schlag and Schlag-Rey, 2002). A prominent example of retinotopic, eye position dependent coding are gain fields as illustrated by several computational models (Zipser and Andersen, 1988; Salinas and Sejnowski, 2001; Pouget et al., 2002). Gain fields describe neurons that show a multiplicative coding of eye position and retinotopic stimulus position, i.e., while the stimulus is placed at exactly the same position on the retina, the neural response to that stimulus depends on the position of the eye in the orbit. Such gain fields have been observed in several parietal areas, such as in LIP (Andersen and Mountcastle, 1983; Bremmer et al., 1997; Boussaoud and Bremmer, 1999) and recent computational modeling demonstrated that gain field properties can be learned using Hebbian learning rules (De Meyer and Spratling, 2011). While most visual areas have neurons with retinotopic receptive fields, it has been reported that some cells in the lateral intraparietal area (LIP) have head-centered stimulus representations (Galletti et al., 1993; Mullette-Gillman et al., 2005). However, the basic mechanisms of visual stability should already act at the retinocentric level. In the static case, i.e., during fixation, gain fields are known to solve the stability problem since they can serve as basis-functions to compute an eye position invariant representation (Pouget et al., 2002). However, in trans-saccadic perception gain-fields would require slightly delayed, but perfect information about eye position: According to the reafferent principle (Von Holst and Mittelstaedt, 1950) changes in the afferent visual pathway that occur during gaze shifts can be compensated by an efference signal from the motor command to ensure perceptual stability. Recent computational studies showed that an instantaneous eye position is not necessary but a slightly delayed one (Teichert et al., 2010). However, there is so far no evidence that this kind of information is available. In particular, a solution of visual stability based on gain fields has recently been challenged by the observation that eye position information in LIP updates late after saccade (Xu et al., 2012), so that the post-saccadic view would be still processed with pre-saccadic eye position information.

We propose a solution that considers two interacting streams of processing, one stream influenced by corollary discharge and the other by eye position. We implemented this concept in a neuro-computational model and first show how both streams interact to produce a remapping of receptive fields. We then link the mechanism of remapping to visual stability by simulating the saccadic suppression of displacement (SSD) paradigm using the same model. In brief, the SSD experiment requires subjects to report if a stimulus has been displaced either to the left or right. In human subjects it has been observed that the threshold for the detection of stimulus displacements is increased when the displacement occurs during a saccade (Bridgeman et al., 1975; Deubel et al., 1996). However, SSD has so far not been explained by any neuro-computational model.

As our neuro-computational model has been already introduced in Ziesche and Hamker (2011), we here follow the approach to accumulate evidence for an already existing model of visual stability, as one of the ultimate goals in computational neuroscience should be to demonstrate that a single computational systems-level model can account for multiple experimental observations. Ziesche and Hamker (2011) introduced this model and showed that it can explain data of peri-saccadic mislocalization of briefly flashed stimuli in total darkness, i.e., the observation that a stimulus, briefly flashed around saccade onset, is not perceived at its flashed location but at a location almost half of the saccade amplitude away in direction of the saccade vector. Keeping the same parameters that have been chosen to match the behavioral data of peri-saccadic mislocalization in total darkness (Ziesche and Hamker, 2011), we show here that the model can also account for predictive remapping and SSD, which are experimentally quite different from flashes in total darkness.

The model relies on two sources of eye position related signals. The first signal encodes eye-in-head position, which may be proprioceptive and may originate in the somatosensory cortex (Wang et al., 2007). This eye position signal is only updated late after saccade, consistent with the data from Xu et al. (2012). Object and eye-in-head position are integrated into a LIP gain field by a subset of LIP cells, referred to as LIP(PC) cells, inspired by the concept of radial basis function (RBF) networks for coordinate transformation (Pouget and Sejnowski, 1997; Pouget et al., 2002). LIP(PC) neurons are organized in a two-dimensional cartesian coordinate system composed of an axis encoding the retinotopic stimulus position and an axis encoding eye position. The multiplicative interaction between retinotopic stimulus position and eye position leads to a local activation blob of gain field neurons. A projection which sums up all activity along the diagonals of the map provides a head-centered representation of the stimulus, at least during fixation.

The second eye related signal used in the model, a corollary discharge signal (Sommer and Wurtz, 2006; Wurtz, 2008) modulates the gain of another subset of LIP cells, referred to as LIP(CD) cells. The initially independent streams merge at the level of LIP and a perceptual decision about stimulus displacement is obtained by a temporal integration of the neural responses in both streams, corollary discharge and eye position modulated cells. Thus, we show that the impairment of perception due to the delayed update of eye position signals observed in LIP (Xu et al., 2012) can be compensated by a corollary discharge signal.

The model explains the reduced sensitivity to stimulus displacement, observed in SSD experiments, by recurrent processing such that the pre-saccadic stimulus affects the neural response of the post-saccadic stimulus. While the pre-saccadic stimulus is processed it affects the processing of the displaced stimulus which could be referred to a masking phenomenon. Predictive remapping not only establishes to connect the pre- with the post-saccadic view but also facilitates recurrent processing to increase the threshold for displacement detection. By suppressing corollary discharge and predictive remapping in the model, we provide for the first time testable predictions how these basic physiological phenomena contribute to visual stability.

## 2. Materials and methods

### 2.1. Simulation of experimental paradigms

**Figure 1:**
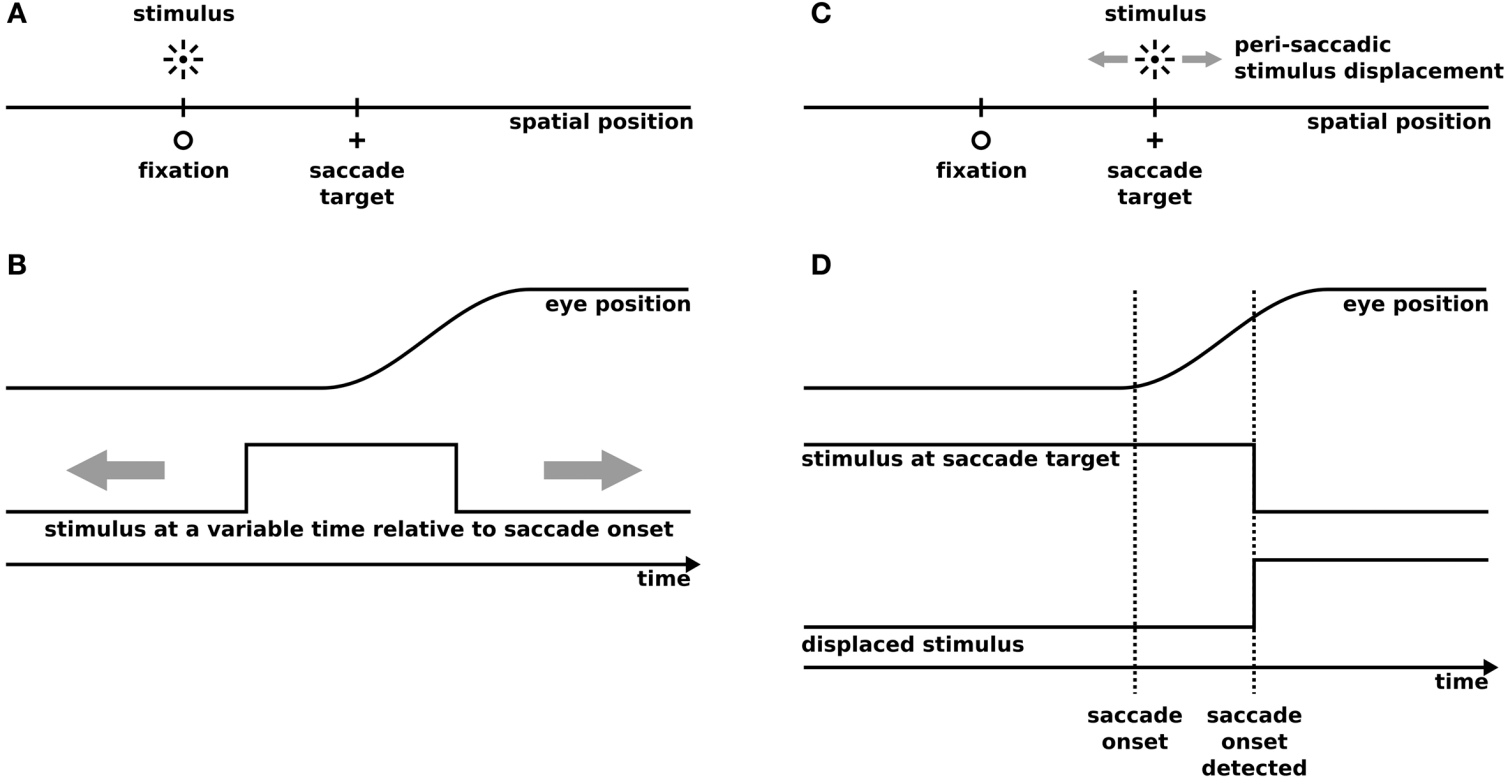
**The simulated experimental paradigms**. **(A,B)** Predictive remapping according to the procedure of Kusunoki and Goldberg (2003). **(A)** The spatial setup of predictive remapping. For simplicity we only simulate a one-dimensional space. We simulate saccadic eye movements from fixation to a saccade target using a saccade amplitude of 14°, while the stimulus is presented at the fixation position (in the future receptive field) or at −14° (present receptive field). **(B)** The temporal setup of predictive remapping. 100 ms flashes are presented at a variable time around the saccade between 500 ms before and 100 ms after saccade onset and the neural response has been recorded and averaged in a time window between 50 and 350 ms after stimulus onset in monkey and model. **(C)** The spatial setup of saccadic suppression of displacement (SSD). We simulate saccadic eye movements from a fixation to a saccade target using a saccade amplitude of 8°. Peri-saccadically the saccade target is displaced in small steps within a range of −4° to +4°. We simulate a stochastic saccadic scatter with a Gaussian distribution (standard deviation: 0.58°) with an undershoot relative to the intended saccade amplitude (mean undershoot: 0.52°) similar to experimental observations (Niemeier et al., 2003). **(D)** The temporal setup of saccadic suppression of displacement. The stimulus at the saccade target is presented for 500 ms before saccade onset. As in real experiments the saccade target displacement is triggered by the detection of the saccade onset determined by an analysis of the acceleration of the eyes, the displacement is set to take place 30 ms after saccade onset. In the gap condition the stimulus is first extinguished and appears after a temporal gap of 250 ms at its displaced position.

Our model has been tested on two well known experimental paradigms: Predictive remapping and SSD (Figure 1). The predictive remapping paradigm investigates the response of the cell toward a probe presented around saccade onset either within its present or FRF. (Figures 1A,B). In SSD tasks, a saccade target is slightly displaced during the eye movement (Figures 1C,D). After saccade, subjects are required to report the direction of the displacement (e.g., left or right). Both experimental setups have been implemented in a one dimensional space using the same spatial and temporal layout as in the experiments.

### 2.2. Computational model

As the model has been published previously where we applied it to explain the mislocalization of briefly flashed stimuli in total darkness (Ziesche and Hamker, 2011) we here only present the coarse structure of the model. The model and its parameters are identical to those reported by Ziesche and Hamker (2011). The difference to the previous study refers only to the different decision process as obtained by the readout of the neural activity described later in Materials and Methods.

**Figure 2:**
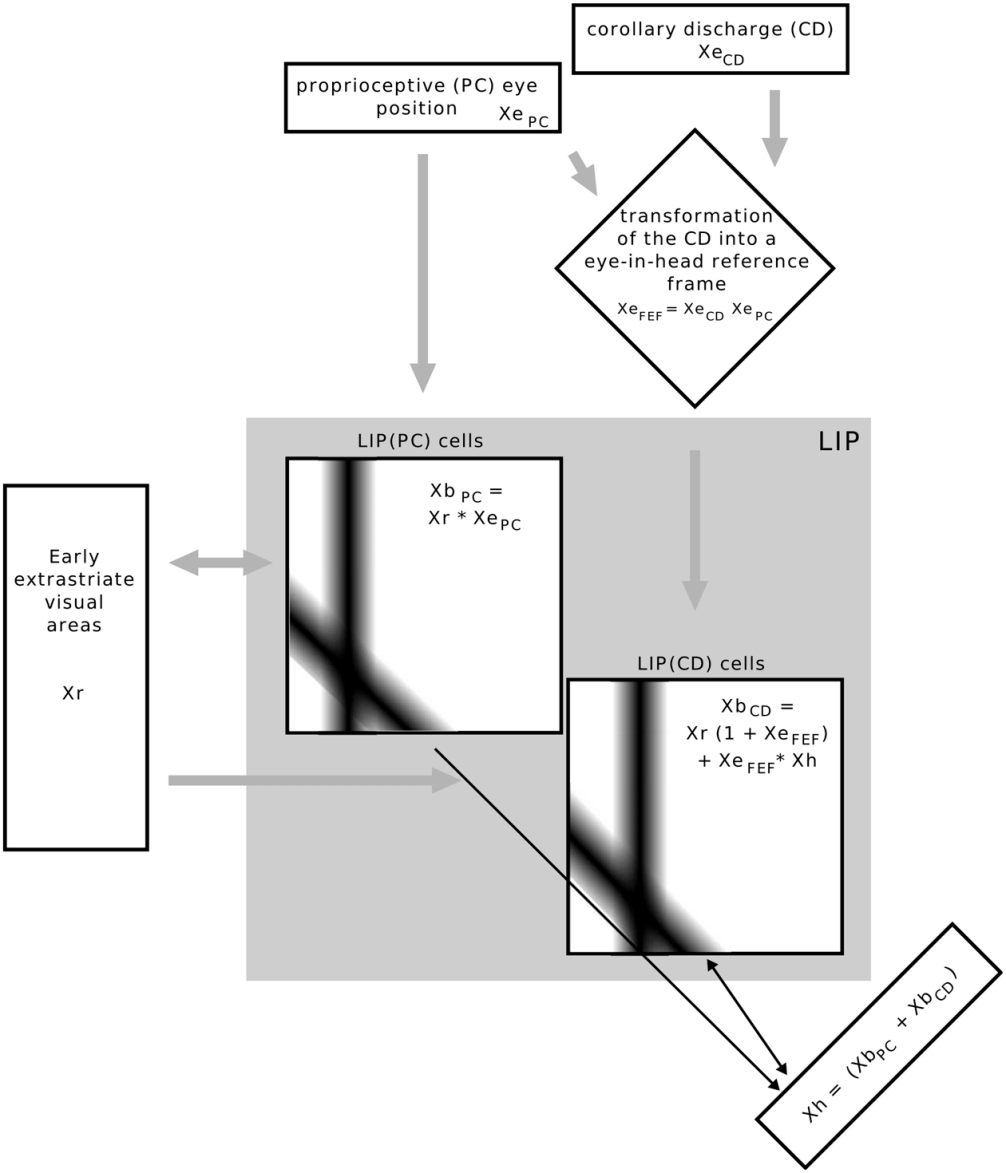
**Overview of the implemented model**. Rectangular boxes indicate a one dimensional space, squared boxes a two dimensional space as determined by their inputs. The equations illustrate how signals are combined in each map, but do not represent the full neural response equations. The bars in the LIP(PC) and LIP(CD) maps illustrate the Gaussian connectivity matrix of a single input neuron to other neurons in the map. Inputs into the whole model are the stimulus position Xr in eye centered coordinates, an eye position signal and a corollary discharge signal that encodes eye displacement. The eye displacement signal is then fed into a gain field to combine the present eye position with corollary discharge, such that one input into LIP is not raw eye displacement, but the anticipatory eye position *Xe*_FEF_, which is only available around saccade, similar to the time course of movement and visuo-movement cells in the frontal eye field. LIP consists of two types of cells which are here organized in different maps. The response of LIP(PC) cells expressed by *Xe*_PC_ is determined by the multiplicative interactions of Xr and *Xe*_PC_. As the connection pattern of both inputs is determined by a Gaussian function an activation blob emerges at those LIP(PC) cells which are tuned to the present stimulus (ordinate) and eye position (abscissa). LIP(CD) cells, expressed by *Xb*_CD_, are also organized by a two dimensional map where the ordinate represents stimulus position and the abscissa the future (post-saccadic) eye position. LIP(CD) cells generally respond to the stimulus position, even without the presence of other eye position-related inputs. However, the gain of those cells that are tuned to the future eye position is increased by *Xe*_FEF_ in the moment around saccade. Essential for the observation of predictive remapping is the additional input *Xe*_FEF_ × *Xh* to LIP(CD) cells. First of all, *Xh* receives its input from LIP(PC) cells such that it encodes the stimulus position in a head-centered reference frame by taking a sum along the diagonals (Pouget et al., 2002). However, in addition *Xh* neurons receive input from LIP(CD) cells, which encodes prior to saccade onset the stimulus position also in a head-centered reference frame, but now with reference to the future eye position. This combination of stimulus position related to the present and future eye position is fed back to LIP(CD) cells and elicits a response at those neurons that are tuned to pre-saccadic visual locations with respect to the CD signal (see Figure 3), as illustrated by the connections matrix in the LIP(CD) map.

The model is focused on area LIP but also relates to some brain areas which project to LIP (Figure 2). It has three different inputs: (proprioceptive) eye position *Xe*_PC_, corollary discharge signal *Xe*_CD_, and retinal object position *Xr*. LIP combines information about stimulus position with two eye related signals, an eye position and a corollary discharge signal. One set of LIP cells are gain field neurons modulated by eye position (*Xe*_PC_) and thus referred to as LIP(PC) cells and another set of cells, LIP(CD) cells, are gain modulated by a corollary discharge signal (*Xe*_CD_). We first explain the characteristics of the retinotopic input map that represents early visual areas such as MT or V4, the eye-related signals, then the two types of LIP cells and their interaction, either via the map of *Xh* or by direct lateral connections. Finally, we explain how the neural activity in the model is read out for a perceptual decision with respect to the direction of stimulus displacement.

We simulate all one-dimensional maps with *n* = 40 neurons and all two-dimensional maps with *n* neurons along each dimension resulting in a total of *n*^2^ neurons for each of these layers. The firing rate of each neuron is computed in a time continuous fashion using an ordinary differential equation (ODE). We simulate a visual field of *v* = 160°, ranging from −80° to 80°.

#### 2.2.1. Retinotopic map *Xr*

A set of *n* = 40 neurons with Gaussian receptive fields covers the whole visual field such that the strength of the neural input of each neuron depends on the distance between stimulus position and RF center. The width of the RF is a function of the eccentricity. The activity of a given *Xr* cell *i* is given by $\tau \frac{d}{dt}r_{i}^{\text{Xr}}=I\left(1+f\left(r_{l,m}^{{\text{Xb}}_{\text{PC}}}\right)\right)-r_{i}^{\text{Xr}}$, where *I* is the input as determined by the Gaussian receptive fields which is gain modulated by the feedback of the response *r*^Xb_PC_^_*l*,*m*_ from the neurons in the *Xe*_PC_ map using a non-linear function *f*. The feedback term is not critical for the results as it only transfers changes in higher areas back to earlier ones, a putative mechanism of attention.

#### 2.2.2. Eye position map *Xe*_PC_

A set of *n* eye position neurons code the present eye position by a Gaussian activation profile. The activity of a *Xe*_PC_ cell *i* is given by $\tau \frac{d}{dt}r_{i}^{{\text{Xe}}_{\text{PC}}}=r_{i}^{{\text{Xe}}_{\text{PC},\text{in}}}-r_{i}^{{\text{Xe}}_{\text{PC}}}$, where *r*^Xe_PC,in_^_*i*_ is the eye position input. We take the assumption that the eye position signal updates only after saccade to its new location, as supported by recent findings (Xu et al., 2012). As it might originate in the primary somatosensory cortex (Wang et al., 2007; Xu et al., 2011), we use the term *Xe*_PC_ to refer to this eye-position signal. However, its exact origin is not fundamentally relevant for the results presented.

#### 2.2.3. Corollary discharge map *Xe*_CD_

A set of *n* corollary discharge neurons code the planned saccade displacement in retinotopic coordinates by a Gaussian activation profile at the eccentricity coding the particular displacement. Corollary discharge might originate in the superior colliculus (SC) and is routed via the frontal eye field (FEF) to visual areas (Sommer and Wurtz, 2004; Sommer and Wurtz, 2006). Thus, a natural assumption is that its time course is similar as reported from single cell recordings of visuo-movement cells in SC and FEF (Sommer and Wurtz, 2004). It increases prior to saccade, is maximally active around saccade onset and then decays. This time course is modeled by a Gaussian rise and a slower Gaussian decay similar as in a previous model (Hamker et al., 2008). The firing rates are again modeled by ODEs.

#### 2.2.4. Eye movement map *Xe*_FEF_

As both streams, the corollary discharge and eye position, are integrated at the level of LIP, eye position and eye displacement have to be transferred into a common reference frame. Inspired by observations of Cassanello and Ferrera (2007), Ziesche and Hamker (2011) showed that a gain field in the FEF can transform eye displacement into eye position (Figure 2), while keeping the time-course of the signal, such that two eye-in-head position signals are available, one phasic signal that anticipates the goal-location of the saccade (*Xe*_CD_) and one tonic signal that encodes the present eye position and updates after saccade (*Xe*_PC_). The transformation is sketched as *Xb*_FEF_ = *Xe*_CD_ × *Xe*_PC_ and the implementation follows the classical basis function map of Pouget et al. (2002), except that it is computed with a time varying input and different equations for the rate coded neurons. *Xe*_FEF_ is now simply the sum along the diagonals in this map.

#### 2.2.5. Basis function map *Xb*_PC_

**Figure 3:**
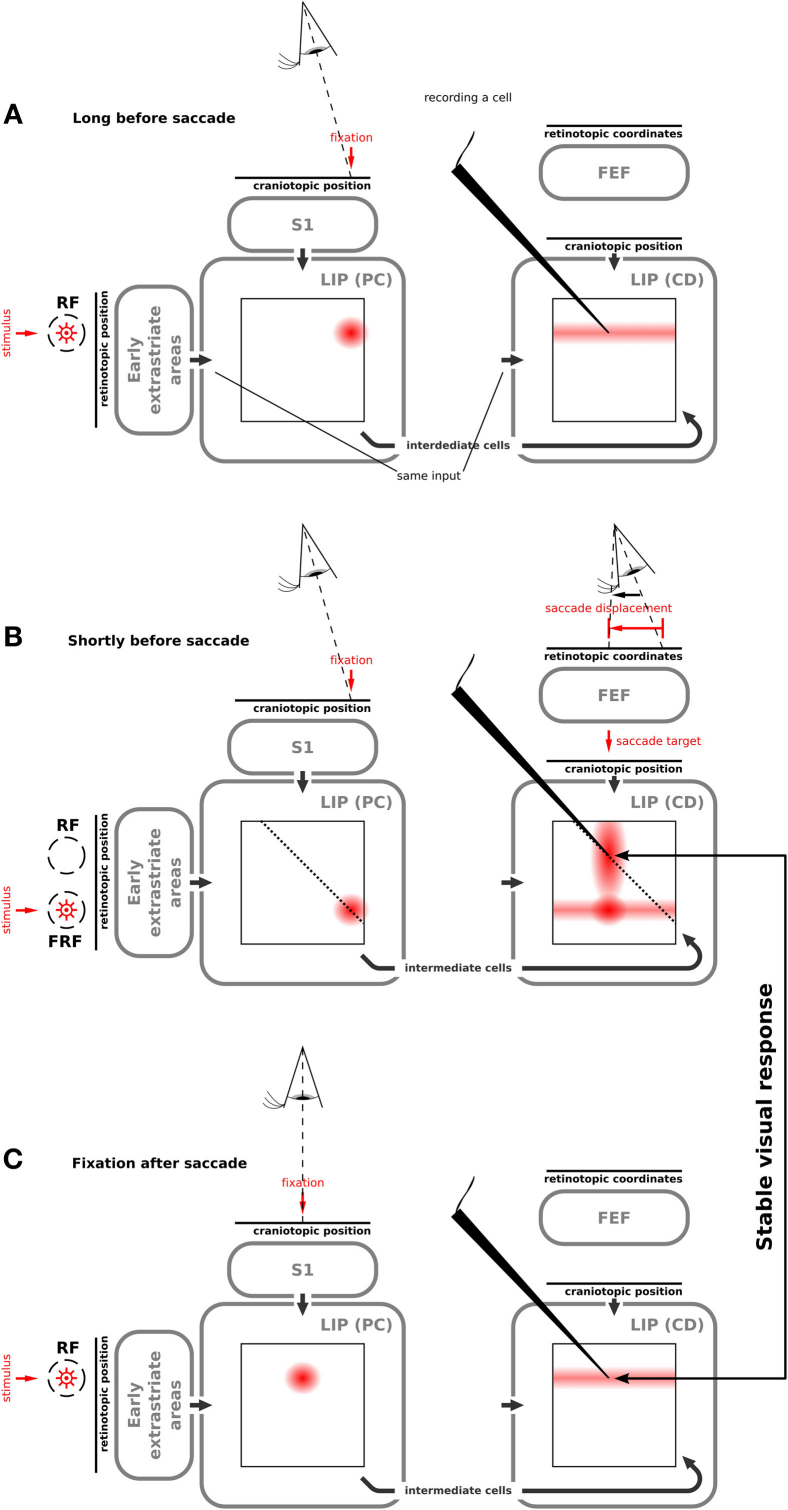
**Illustration of how our model produces predictive remapping behavior**. Please refer to Movie S1 that shows the model simulation. We show the activity of the two LIP cell types in different maps. On the vertical axis they code retinal stimulus position; on the horizontal axis head-centered proprioceptive (PC) signal in LIP(PC) and head-centered corollary discharge (CD) in LIP(CD). Both maps are connected via the intermediate cells. The wedge points to a LIP(CD) cell which shows a predictive remapping response. **(A)** Network activities long before the saccade when a stimulus is presented in the present receptive field. LIP(PC) shows a joint representation of the visual signal and the proprioceptive signal, which encodes the pre-saccadic eye position. The “predictive remapping” LIP(CD) cell responds to the stimulus presented within its receptive field. **(B)** Network activities shortly before the saccade when the stimulus is presented in the future receptive field of the “predictive remapping” cell. In LIP(PC) a cell responds, for which the stimulus is in its normal receptive field. LIP(CD) similarly responds to this stimulus, however, due to the corollary discharge, which encodes the saccade target, the visual response in LIP(CD) is partially increased. Furthermore, the activity from LIP(PC) is projected along the dashed lines into LIP(CD) using the connection via the intermediate cells. This activity interacts with the corollary discharge leading to activity at the “predictive remapping” cell. **(C)** After the saccade, the receptive field of the “predictive remapping” cell has moved to the position of the future receptive field from the previous step. In LIP(CD) the evoked response includes the “predictive remapping” cell. Hence this cell responds to the same stimulus as shortly before the saccade. It shows “visual stability.”

The eye position related stream of stimulus position describes the response of LIP(PC) cells. The neurons are organized as a basis function map *Xb*_PC_, which follows the classical basis function map of Pouget et al. (2002), except that it is computed with a time varying input and different equations for the rate coded neurons. The transformation is sketched as *Xb*_PC_ = Xr × *Xe*_PC_. Basically, a two-dimensional map of *n* × n neurons is created from the two single dimension population inputs (eye position and stimulus position) which mimics eye position dependent responses in LIP. Given a particular stimulus and eye position, a single activity hill in *Xb*_PC_ emerges which is maximal at the intersection between both inputs (Figure 3A).

#### 2.2.6. Gain modulation map *Xb*_CD_

The gain modulation map *Xb*_CD_ describes the response of LIP(CD) cells. It is composed of *n* × n neurons and integrates the CD signal with stimulus position. Different from *Xb*_PC_ the cells in *Xb*_CD_ are simply gain modulated by the CD signal and thus would not be classified as an eye position gain field. The cells always respond to stimuli in their RF even in the absence of a CD signal long before a saccade (Figure 3A) indicated by the term *Xr* × (1 + *Xe*_FEF_) in Figure 2, which is typical for attentional gain modulation (Zirnsak et al., 2011a). In addition to this feedforward input, mutual interactions between LIP(PC) and LIP(CD) cells are essential for predictive remapping. Two slightly different model versions have been developed (Ziesche and Hamker, 2011), while both can account for the peri-saccadic spatial mislocalization of briefly flashed stimuli in total darkness (Van Wetter and Van Opstal, 2008): one model that avoids any non-retinocentric stimulus representation where LIP(PC) and LIP(CD) cells communicate via lateral connections and another where LIP(PC) and LIP(CD) cells communicate via intermediate cells (Xh) whose receptive fields are head-centered (Figure 2). The communication within the maps has been implemented to show predictive remapping in LIP(CD) cells: The maps in LIP(PC) and LIP(CD) will be read out by a projection of all cells along the diagonals to integrate the evidence of stimulus position relative to the present eye position and relative to the intended saccade target position into a single population of Xh cells. As the population of Xh cells projects back into LIP(CD) along the diagonal direction and this feedback signal is multiplicatively combined with the anticipatory saccade target *Xh* × *Xe*_FEF_ (see also Figure 2), LIP(CD) cells do respond to stimuli in their FRF given the anticipatory CD signal (Figure 3B). Thus, our model does not need all-to-all connections among LIP cells as required in a previous model of remapping (Quaia et al., 1998), it only requires a short range Gaussian connectivity profile from each input (Figure 2): *Xe*_FEF_ in the vertical direction, Xr in the horizontal direction and *Xh* in the diagonal direction.

#### 2.2.7. Intermediate cells *Xh*

One of the two developed models uses an explicit representation of stimulus position in a head-centered reference frame at the level of *Xh*. A set of *n* intermediate cells receive their input from both streams: *Xb*_CD_ and *Xb*_PC_ by collecting all activity along the diagonals, which ensures a head-centered reference frame (Pouget et al., 2002). Thus, these cells merge information about stimulus position relative to eye position and the anticipatory component which is stimulus position relative to the predicted future eye position. Both are not identical around saccade.

### 2.3. Decoding of neural activity for decision making

The SSD paradigm requires the model to report “forward displacement” and “backward displacement” decisions instead of absolute spatial positions as required by the localization of briefly flashed stimuli. Rather than taking a particular snapshot in time we use a layer of decision neurons that accumulate evidence over time and compete for the final decision, but compared to the previous approach (Ziesche and Hamker, 2011) require only two cells (forward and backward displacement). In the model without intermediate cells, the decision process receives input from both, LIP(CD) and LIP(PC). In the model with intermediate cells their activity is used as input for the decision process. Thus, the neural response is fed into a diffusion model with decision neurons (Usher and McClelland, 2001; Hamker, 2007). Besides being justified by empirical studies (Kiani et al., 2008; Stanford et al., 2010) this approach has the advantages of accumulating varying evidence over time and allowing stochastic simulations.

In order to compute a final response of the model with respect to forward or backward displacement in the SSD paradigm a temporal integration composed of the following steps is applied:

1. In the head-centered model the input *I*^DP^ to the decision process consists of the firing rates of Xh, i.e., *I*^DP^_*i*_ = *r*^Xh^_*i*_. In the non-head centered model a similar input is generated by
  (1) $$I_{i}^{\text{DP}}=\sum_{lm}w_{ilm}^{{\text{Xb}}_{\text{PC}},\text{DP}}r_{lm}^{{\text{Xb}}_{\text{PC}}}+\sum_{lm}w_{ilm}^{{\text{Xb}}_{\text{CD}},\text{DP}}r_{lm}^{{\text{Xb}}_{\text{CD}}}+$$
  where the connection weights are also similar to those of Xh:
  (2) $$\begin{array}{l} \;w_{ilm}^{{\text{Xb}}_{\text{PC}},\text{DP}}=K^{{\text{Xb}}_{\text{PC}},\text{DP}}\exp\frac{-\|i-l-m{\|}^{2}}{{\left(\sigma^{{\text{Xb}}_{\text{PC}},\text{DP}}\right)}^{2}}\\ \left(\sigma^{{\text{Xb}}_{\text{PC}},\text{DP}}=15.0,\;K^{{\text{Xb}}_{\text{PC}},\text{DP}}=0.035\right) \end{array}$$
  (3) $$\begin{array}{l} \;w_{ilm}^{{\text{Xb}}_{\text{CD}},\text{DP}}=K^{{\text{Xb}}_{\text{CD}},\text{DP}}\exp\frac{-\|i-l-m{\|}^{2}}{{\left(\sigma^{{\text{Xb}}_{\text{CD}},\text{DP}}\right)}^{2}}\\ \left(\sigma^{{\text{Xb}}_{\text{CD}},\text{DP}}=15.0,\;K^{{\text{Xb}}_{\text{CD}},\text{DP}}=0.02\right) \end{array}$$
2. The start time of the accumulation process is set to 28 ms after saccade offset for conditions where the displaced stimulus reappears during the saccade and 60 ms after stimulus onset when the displaced stimulus reappears after saccade offset. We chose not to couple the accumulation onset to stimulus onset for peri-saccadic stimuli since peri-saccadic detectability of stimuli is suppressed (Volkmann et al., 1978). The timing of 28 ms after saccade offset is motivated by assuming that perceptual decision primarily relied on the post-saccadic view (while considering neural latency).
3. The position information encoded in the input *I*^DP^ is decoded using template matching with precalculated templates with a step size of 0.5°. Template matching is done using correlation. The match *m*_*c*_ of the template *t*^*c*^_*i*_ representing a stimulus at position *c* with neurons *i* is $m_{c}=\sum_{j}I_{j}^{\text{DP}}t_{j}^{c}$. The spatial resolution of the decision neurons equals that of the templates.
4. We introduce noise by transforming the firing rate into a Poisson spike train. To be more specific, one time step of the input *m*_*c*_ (the template match from the previous step) is equivalent to *n* time steps of the spiking neuron ${\tilde{m}}_{c}$ (*n* = 20 is the bin size). Spiking is simulated in the simplest way: In each of the *n* time steps the neuron spikes if and only if *m*_*c*_ > *R* × *s*_max_ where *R* is a random number between 0 and 1. The spiking activity of the neuron is *s*_max_ = 1 while the non-spiking activity is 0. Then the spike train is averaged for accumulation in the decision neurons.
5. The previous step provides us evidence for the presence of a stimulus at each spatial position with a resolution of 0.5°. At this step, we collect all these evidences into two evidences *m*_*f*_ and *m*_*b*_, one for forward and one for backward displacements using the pre-saccadic stimulus position *c*_pre_ as a decision border: *m*_*f*_ = ∑_c ≥ *c*_pre__ *m*_*c*_ and *m*_*b*_ = ∑_c ≤ *c*_pre__ *m*_*c*_
6. We implement a competition between the decision neurons by subtracting each input from the other: *m*_*f*_ = *m*_*f*_ − *m*_*b*_ and *m*_*b*_ = *m*_*b*_ − *m*_*f*_.
7. Accumulating decision neurons are implemented as in Hamker (2007). The ODE of each of the two decision neurons *d*_*f*_ and *d*_*b*_ is: $\tau^{\text{DN}}\frac{t}{dt}d_{f/b}(t)=m_{f/b}$ with time constant τ^DN^ = 50 ms. Each decision neuron *d*_*c*_ is initialized with a baseline firing rate of 0.1 before the decision process begins. A decision is made once one of the neurons reaches the threshold *d*_thresh_ = 0.3 to 0.9 (the threshold is varied in some of the simulations). If none of the neurons reaches this threshold after 100 ms the neuron with the highest activity at that time wins (see Kiani et al., 2008).
8. This whole process is repeated 20 times and then averaged over all trials for each condition, i.e., saccade amplitude, motor error, displacement. Thereby, we calculate the average number of “forward displacement” responses.

### 2.4. Simulation of saccadic eye movements

We reimplemented the saccade generator from Van Wetter and Van Opstal (2008) to simulate the spatiotemporal trajectory of a saccade. The simulation of the suppression of displacement paradigm requires to account for stochastic scatter in order to compute realistic trial to trial variations. In addition, we chose on average a small undershoot in amplitude, as often observed in experimental data (Niemeier et al., 2003). Thus, from each experimentally required “correct” saccade we substract the scatter sampling from a Gaussian distribution *N*(μ = 0.52°, σ = 0.58°). In addition to a variation of the saccade amplitude, we have to specify in how far the CD signal anticipates the final saccade amplitude. We assume that the saccadic scatter arises on the motor side, thus it is not reflected in the CD signal, but the CD signal reflects the average undershoot (Collins et al., 2009).

## 3. Results

### 3.1. Predictive remapping in the model

All simulations have been done with the whole systems-level model (Figure 2). We initially focus on explaining the implemented mechanisms that result in the predictive remapping responses of LIP(CD) cells. Although the particular type of implementation of predictive remapping is novel, it has been explicitly built into the model. Long time before saccade (Figure 3A), a stimulus leads to visual responses in LIP(PC) and LIP(CD). Shortly before saccade (Figure 3B, see also Movie S1), the eye is still fixating at the same position (Figure 3B, left) while a saccade plan is set in place (Figure 3B, right) that in turn will change the spatial selectivity of the LIP(CD) cell being recorded from. The saccade plan is fed as a saccadic displacement vector into the model FEF mimicking the CD signal from the SC reported by Sommer and Wurtz (2008). Due to eye position gain fields in FEF (see Ziesche and Hamker, 2011), the corollary discharge encodes the intended saccade target in LIP(CD). The crucial component for predictive remapping to occur is that the visual response from LIP(PC) elicited by the probe in the FRF is fed into LIP(CD) via the intermediate cells. The connection patterns are such that all activity in LIP(PC) which occurs along the diagonal lines are fed to those cells in LIP(CD) that are located along the diagonal (Figure 2). In this particular case the activity along the dashed line in LIP(PC) is summed up and projected to all LIP(CD) cells along the dashed line (Figure 3). In LIP(CD) this input interacts multiplicatively with the corollary discharge which is fed into LIP(CD) along vertical lines. In effect, the recorded cell in LIP(CD) responds to the probe in the FRF. This response is what would have been expected from the classical formulation of predictive remapping. However, the Movie S1 illustrates that cells with receptive fields in between the RF and FRF can also show predictive remapping responses. The exact spatio-temporal distribution of predictive remapping responses depends on a number of parameters that influence the dynamics in the network. Theoretically, the model can be tuned toward more sharply tuned, separated activity hills with less activation of cells having receptive fields in between the classical and future RF. However, this rather broad activation can be considered as an implicit prediction of the model, since it was helpful in fitting the model to the behavioral data of peri-saccadic mislocalization of briefly flashed bars in total darkness (Ziesche and Hamker, 2011). So far only a few cells in the FEF but not in LIP have been analyzed to explore the spatial distribution of remapping (Sommer and Wurtz, 2006). Note, that there is also an increased visual response in LIP(CD) for those cells which receive the activity from the corollary discharge [illustrated by a blob on the horizontal line of activity in LIP(CD)]. Some time after the saccade (Figure 3C), the corollary discharge signal has decayed and the proprioceptive eye position signal has updated to encode the post-saccadic fixation. Now, the normal receptive fields in LIP(CD) are restored.

**Figure 4:**
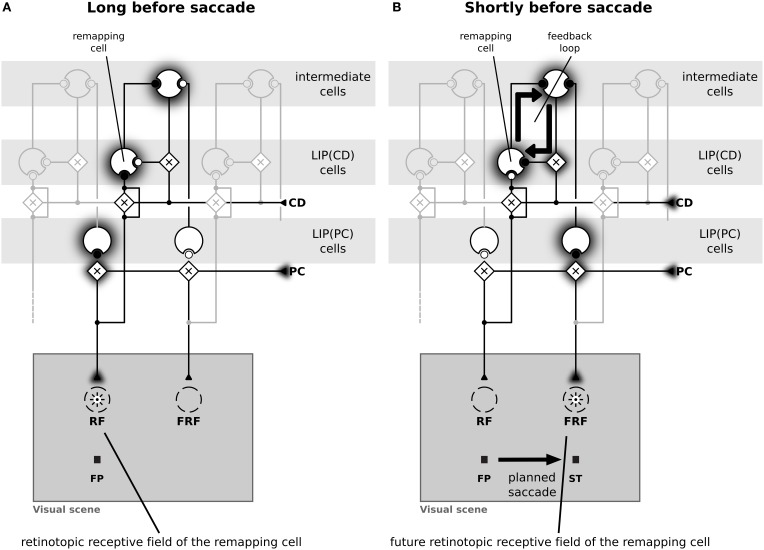
**Schematic diagram of the neural circuit that implements predictive remapping in the model LIP**. This diagram details the connections from Figure 2 but shows only relevant connections in high detail and does not visualize all other projections. “Predictive remapping” refers to the phenomenon that some visual cells with retinotopic receptive fields (RFs) transiently respond to stimuli in their post-saccadic receptive fields (future receptive field, FRF) shortly before an upcoming saccadic eye movement. We propose a model of the lateral intraparietal area (LIP) which is composed of two types of retinotopic visual cells. LIP(PC) cells, which are gain modulated by a proprioceptive (PC) eye position signal, and LIP(CD) cells, which are gain modulated by a corollary discharge (CD). Thus, each LIP(PC) cell only responds to a specific combination of retinotopic stimulus position and gaze direction. In contrast, the receptive fields of LIP(CD) cells show a gain increase for certain saccade targets encoded by the corollary discharge. Black circles indicate active and white circles inactive synaptic connections. **(A)** During fixation both, an LIP(PC) and an LIP(CD) cell respond to a stimulus in their retinotopic receptive field. Long before saccade the CD signal is inactive. **(B)** A predictive remapping cell, here a LIP(CD) cell, responds when the stimulus is presented shortly before saccade onset at the future receptive field. In our model, this occurs as LIP(PC) cells, which have their RF located at the location of the FRF of the considered LIP(CD) remapping cell, project to intermediate cells. Their activity, but only in combination with a spatially selective activation of a CD signal drives the remapping cell.

Figure 4 illustrates the implemented mechanism of remapping by showing the relevant connections in detail and leaving out all other ones for a more clear illustration. Figure 4A shows a LIP(CD) remapping cell that responds to a visual stimulus placed within its RF. The same neuron also responds to a stimulation of its future RF (Figure 4B). Rather than by an immediate feedforward connection, the remapping response is elicited, given the presence of a corollary discharge, by a feedback from intermediate cells who in turn receive their input from LIP(PC) cells. The remapping response does not necessarily require an explicit representation of the intermediate cells that encode the stimulus in a head-centered reference frame, the input into LIP(CD) cells can also arise directly from LIP(PC) cells. While, as shown later, both model versions can explain predictive remapping, the model version with intermediate cells has an additional important characteristic relevant for explaining SSD, which is its recurrent loop between LIP(CD) cells and intermediate cells, as this loop stabilizes the pre-saccadic representation during the saccade (Figure 4B).

**Figure 5:**
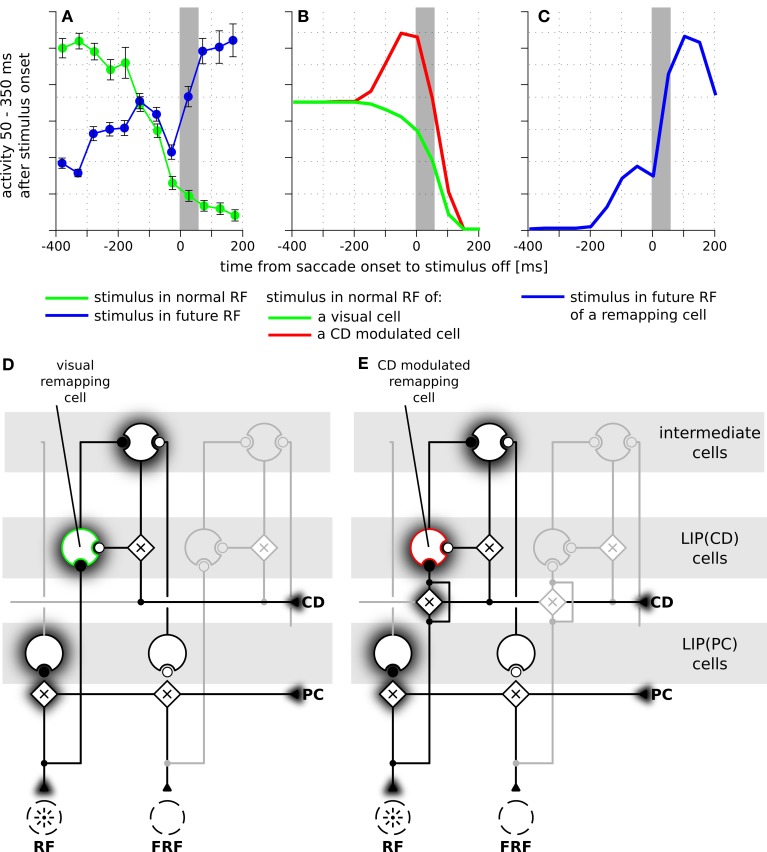
**The time courses of predictive remapping of cells in real and simulated LIP**. The y-axis shows the neural response in the interval from 50 to 350 ms after the appearance of the stimulus aligned to saccade onset. The stimulus was visible for 100 ms. **(A)** Experimental data replotted from Kusunoki and Goldberg (2003). Shown are the averaged responses of 36 visual cells in LIP. Error bars are standard errors. The gray area indicates the duration of a saccade. **(B)** Responses of LIP(CD) cells around saccade (14° amplitude) when a stimulus is presented in the receptive field of the neuron. Green line: responses of a purely visual cell. Red line: responses of a visual cell with a pre-saccadic gain increase. **(C)** Blue line: responses where the stimulus is shown in the future receptive field of the recorded cell. This response is the same for both cell types of panel **(B)** and shows a predictive response. **(D)** Illustration of the connections of a purely visual cell. Its feedforward input from the stimulus is not influenced by the corollary discharge (CD), however, it receives CD-dependent input from the intermediate cells. **(E)** Illustration of the connections of a visual cell with a pre-saccadic gain increase. Its feedforward input from the stimulus increases its gain due to the influence of the CD signal. It can also be driven via the CD-mediated input from intermediate cells (compare Figure 2). Note, this cell does not respond to the planned eye movement in total darkness, as it requires a visual stimulation or at least activity in the intermediate cells.

**Figure 6:**
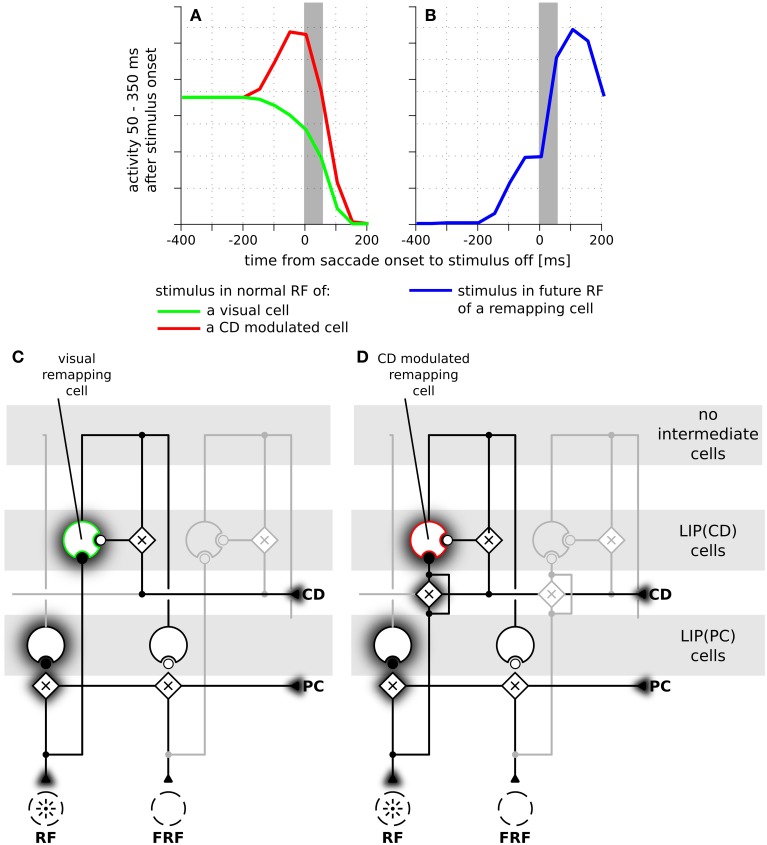
**The time course of predictive remapping in the model without intermediate cells**. **(A)** Responses of LIP(CD) cells around saccade (14° amplitude) when a stimulus is presented in the receptive field of the neuron. Green line: responses of a purely visual cell. Red line: responses of a visual cell with a pre-saccadic gain increase. **(B)** Blue line: responses where the stimulus is shown in the future receptive field of the recorded cell. **(C)** Illustration of the connections of a purely visual cell. Its feedforward input from the stimulus is not influenced by the corollary discharge (CD). Different to the model with intermediate cells it receives CD-dependent lateral input directly from another LIP(PC) cell without any further delay. **(D)** Illustration of the connections of a CD modulated cell.

Having explained how LIP(CD) cells in our model show predictive remapping behavior, we now provide further insight into the model and qualitatively compare it to data from area LIP. In a typical experimental paradigm for studying predictive remapping behavior a stimulus is presented at various times around the saccade in the RF and the FRF of a recorded cell. Figure 5A shows experimental data recorded from LIP cells with predictive remapping behavior (replotted from Kusunoki and Goldberg, 2003). LIP(CD) cells in our model exhibit a comparable predictive remapping behavior (Figures 5B,C). Note that in the model's LIP as presented in Figure 4 all LIP(CD) cells are gain modulated by the corollary discharge and thus show a pre-saccadic enhancement (Bremmer et al., 2009). However, since predictive remapping is also observed for cells without pre-saccadic enhancement (Kusunoki and Goldberg, 2003), we present time courses for the responsiveness to stimuli which are presented in the (normal) RF for a purely visual and visual cell with pre-saccadic enhancement (Figure 5B). The different connectivity for the two types of LIP(CD) cells are shown in Figures 5D,E where the feedforward gain of the cell in Figure 5D is not modulated by the corollary discharge. Since no study has systematically analyzed the remapping of visual cells in LIP with a pre-saccadic enhancement, our model data (dashed line in Figure 5B) serves as a prediction for future experiments. As the difference between the two types of LIP(CD) cells does not affect the predictive responses, we only show one curve for the predictive remapping behavior (Figure 5C). Similar to the experimental data, the simulated cell starts to respond about 150 ms before the saccade to a stimulus which will only be in its RF after the saccade. This predictive remapping response in LIP(CD) cells can be equally well realized without the layer of intermediate cells using a lateral connection from LIP(PC) and LIP(CD) cells (Figure 6).

### 3.2. Saccadic suppression of displacement

In order to investigate the influence of predictive remapping on visual stability across eye movements we operationalize visual stability using the SSD paradigm. Experimental studies revealed that small displacements of the saccade target stimulus are hardly noticed. When forced to make a decision between only two alternatives (forward or backward displacement relative to saccade direction) subjects are uncertain and make more errors as becomes evident by a flat psychometric function which plots the decision of the subject (e.g., the percentage of forward displacements) over the displacement. The exact degree of uncertainty varies across individual subjects (Bridgeman et al., 1975; Deubel et al., 1996; Collins et al., 2009; Ostendorf et al., 2013). However, when the target stimulus is initially extinguished and reappears after a delay of 250 ms at its displaced location, human subjects can detect the displacement surprisingly well as expressed by a steep psychometric function (Deubel et al., 1996).

**Figure 7:**
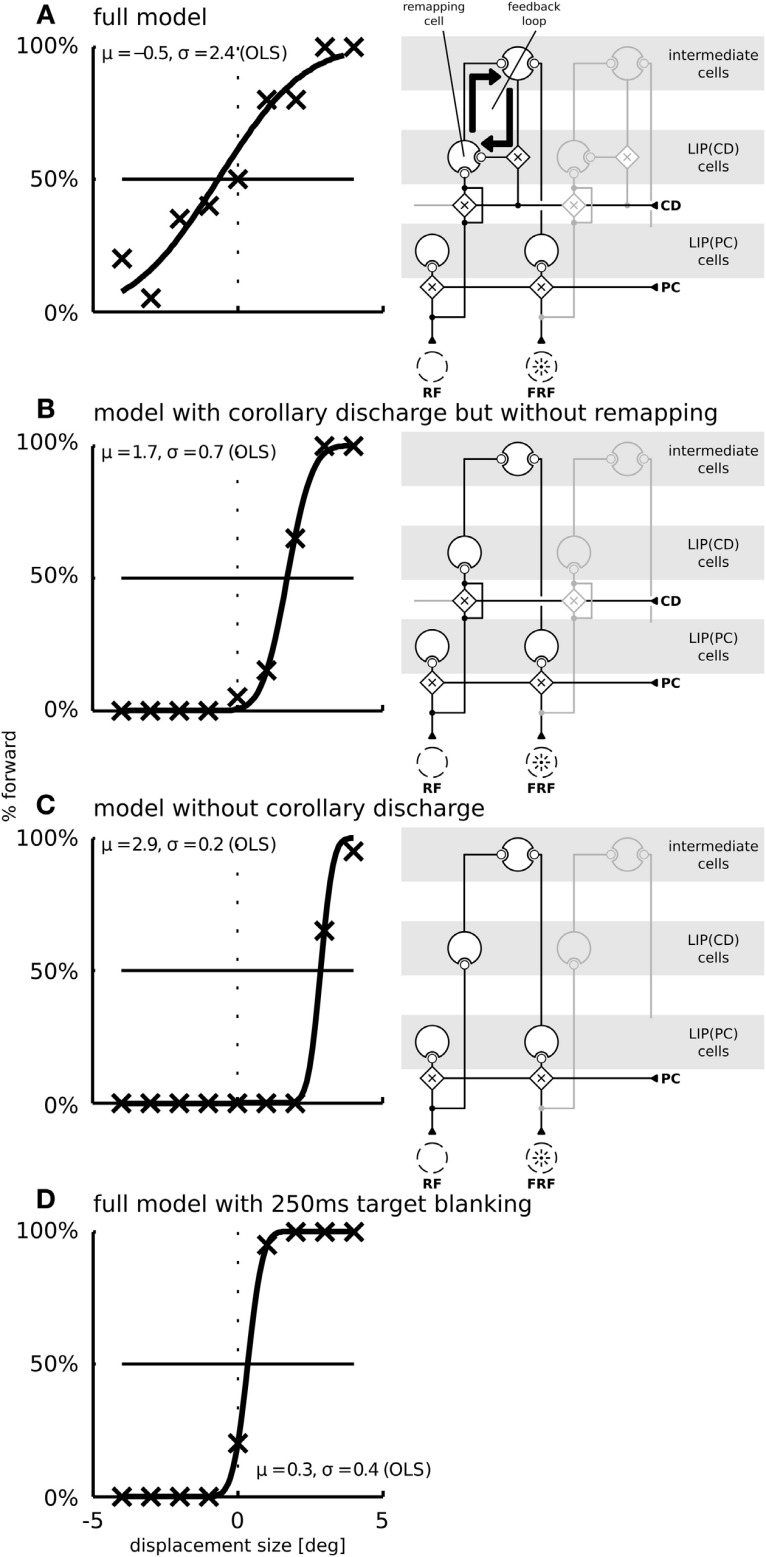
**The function of predictive remapping for visual stability measured by saccadic suppression of displacement (SSD)**. Shown are the model performances in the SSD paradigm for three different model versions. The psychometric plots show the percentage of “forward” replies depending on stimulus jump size. Crosses are simulated data points (averaged over 20 trials per conditions); black lines are cumulative Gaussian distributions which are fitted using ordinary least squares. Parameters of the fitted distributions are shown in each plot. The fitting parameter σ expresses the uncertainty and μ a potential bias in the decision. The right side of each panel depicts which parts of the model are disabled in those simulations. **(A)** In the full model, including corollary discharge and predictive remapping, the response is unbiased. Furthermore, the psychometric curve is shallow, indicating a high threshold for the detection of small stimulus jumps. **(B)** When corollary discharge remains enabled, but predictive remapping responses are disabled [by disabling the feedback to LIP(CD)] the model detects stimulus displacements well. In addition, the response is biased such that displacements of 1.7° are perceived as stable. **(C)** When the corollary discharge signal is disabled, the model has a similar displacement detection performance as in **(B)**, but with an even larger response bias of about 3°. **(D)** When we introduce a 250 ms gap before the stimulus reappears at the displaced position, the displacements are detected well (blanking effect).

We focus our analysis exactly on this qualitative difference and performed simulations for three different models: The full model as described above (Figure 7A), a model without predictive remapping (Figure 7B), and a model without corollary discharge (Figure 7C). The full model shows a typical SSD behavior: The psychometric response curve has a shallow slope, indicating that small target displacements are not perceived well. This diminished detection of displacements is not due to a reduced processing of stimuli but due to the neural trace stemming from the pre-saccadic stimulus. As a control that the steepness of the response curve of the full model (Figure 7A) indeed shows an SSD effect we replicate the well-known, but puzzling blanking effect (Deubel et al., 1996), where the stimulus appears much after the end of the saccade. Such a blanking period of 250 ms restores the displacement detectability also in our model (Figure 7D). According to the model, this blanking effect occurs since the activity traces elicited by the pre-saccadic stimulus have ceased and the proprioceptive eye position signal has updated to its correct, post-saccadic value. In addition, the corollary discharge signal has decayed. Thus, the localization of the displaced stimulus depends solely on the proprioceptive eye position and retinal signals which both are veridical at this time. Thereby, stimulus displacements at this time are detected well.

The model without predictive remapping (Figure 7B) is realized by disabling the feedback from the intermediate cells to LIP(CD) (see the model illustration on the right side of Figure 7B). It predicts two effects on the simulated behavioral outcome: The response is biased toward negative target displacements, i.e., against saccade direction, indicated by the rightward shift of the psychometric function. Moreover, the response curve is steeper which indicates a higher sensitivity. To understand why disabling predictive remapping has this effect, it is necessary to examine the dynamics of the full model, where the perceived target displacement is initially influenced by two main factors (we later discuss a third factor): (1) The saccadic eye movement leads to a movement of the target stimulus on the retina in the opposite saccade direction, i.e., it gives evidence for a negative target displacement (rightward shift of the psychometric function). (2) Predictive remapping distorts the stimulus representation in saccade direction, i.e., it gives evidence for a positive target displacement, since the corollary discharge, which drives the remapping, acts like an anticipatory eye position signal. When remapping is removed, we reduce the evidence for a forward displacement, which explains the bias (Figure 7B).

**Figure 8:**
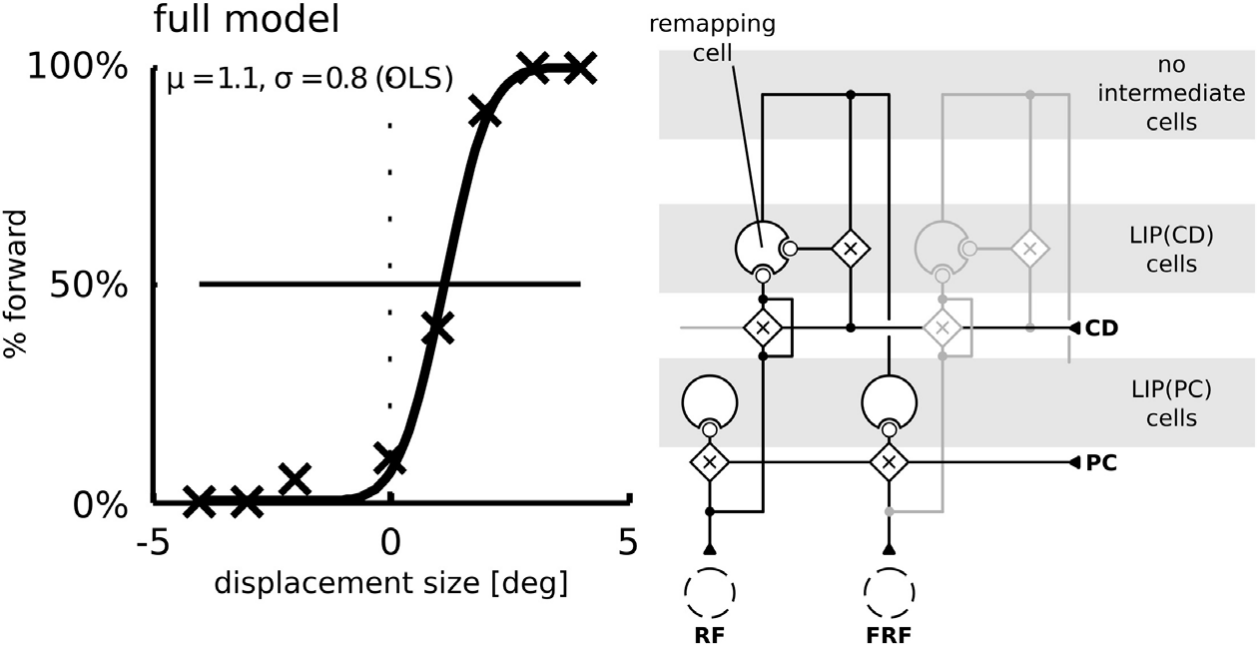
**Simulation of the saccadic suppression of displacement (SSD) paradigm using the model without intermediate cells**. The LIP(CD) cell is modulated by lateral input from LIP(CD) cells. Compared to the model with intermediate cells (Figure 7) the typical flat psychometric response function is missing indicating only little suppression of displacements.

To understand why the psychometric function is steeper in the model without remapping we have to consider a third factor. (3) Remapping establishes a feedback loop. Predictive remapping originates from the lateral projection of LIP(PC) and LIP(CD) via the intermediate cells to LIP(CD) which is modulated by a corollary discharge and is stabilized in the recurrent loop of LIP(CD) to the intermediate cells and back to LIP(CD). Such stabilizing feedback loop has been demonstrated in Figure 3B: Shortly before the saccade the stimulus representation in LIP(PC) is correct since neither the visual signal nor the proprioceptive eye position signal have started updating yet. All network activities along the dashed lines in LIP(PC) and LIP(CD) represent the same stimulus position (in a head-centered reference frame, which is used for the perceptual readout). Since the predictive remapping response is also on the dashed line, it represents the same (correct) stimulus position. Stabilizing the pre-saccadic network activity leads to reduced effects of peri-saccadic stimulus displacements, which explains the SSD effect. If this feedback by remapping is turned off, the stabilizing effect does not occur and peri-saccadic stimulus displacements are detected well. However, this increase in sensitivity for the model without predictive remapping comes with the cost of a bias that occurs by the missing anticipatory component. If we keep predictive remapping in place, but implement it just by a lateral projection directly from LIP(PC) and LIP(CD) without the intermediate cells in between and thus without the recurrent loop of LIP(CD) to the intermediate cells and back to LIP(CD) we also do not observe the typical SSD effect (Figure 8). Thus, predictive remapping together with a peri-saccadic stabilization of the pre-saccadic representation are the crucial ingredients of perceptual stability.

Finally, if the corollary discharge itself is turned off (Figure 7C), the model predicts a large response bias. This is because, similar as for remapping, the corollary discharge signal leads to a distortion of the stimulus representation in saccade direction. Thus, when corollary discharge is disabled, there is an increased response bias toward negative displacements. At the same time, the steepness of the response curve is similar to the model without remapping (in panel **B**) as without corollary discharge the stabilizing feedback loop is interrupted.

**Figure 9:**
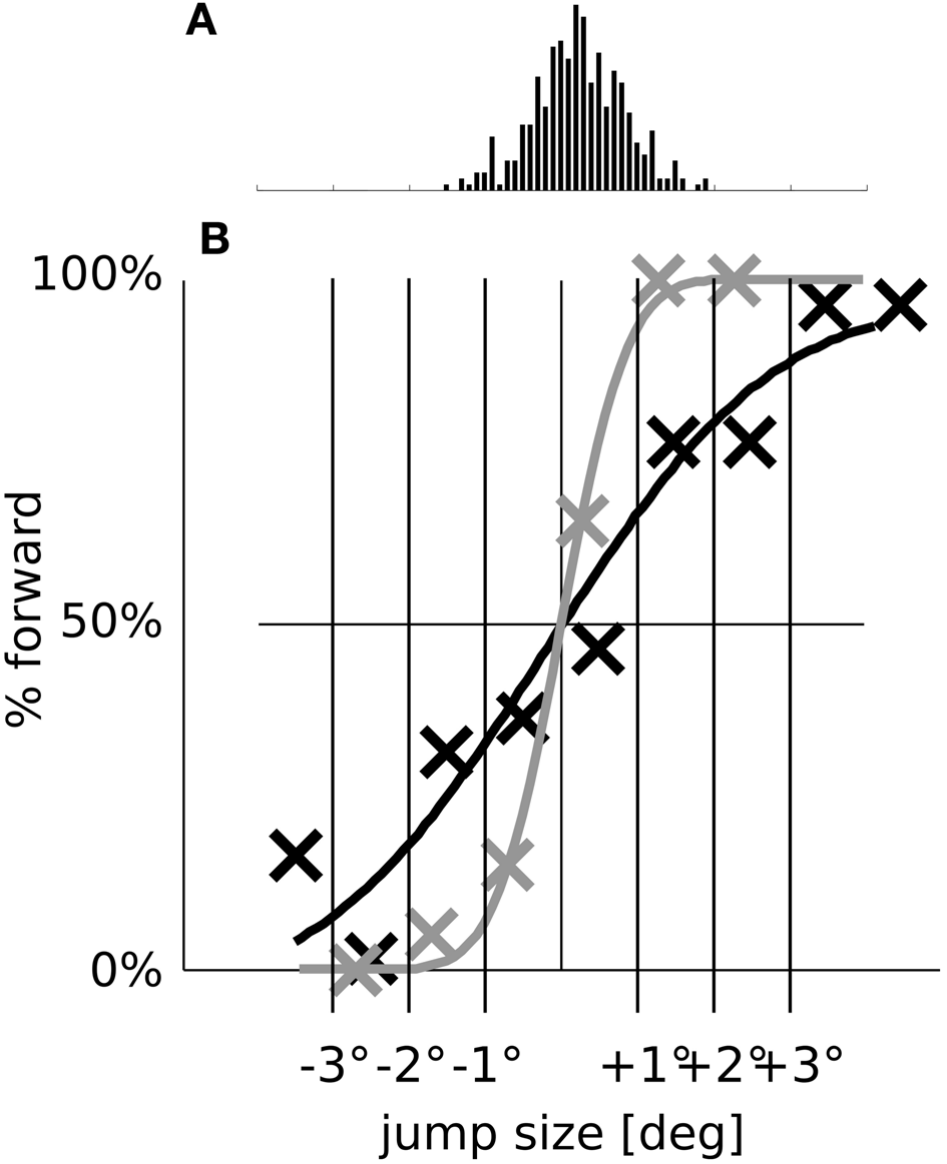
**The SSD effect in relation to saccadic scatter**. **(A)** Histogram of the saccade endpoint with removed bias (in the simulations, the saccades undershoot by 0.52° on average). **(B)** For better comparison, we replot the psychometric detection curves of the full model (dark line) and of the model without remapping (gray line) from Figures 7A,B with the biases removed.

In order to further reveal the effect of predictive remapping we show in Figure 9B the psychometric detection curves from Figures 7A,B in a different fashion on top of each other and without the bias. We removed the bias by shifting the psychometric curves such that they pass through 50% detection performances for 0° target displacements. For comparison, Figure 9A shows a histogram of the saccadic scatter from eye movement simulations (where the bias was removed as well). It can be observed that without predictive remapping, the stimulus jump detection curve is steep and trials with a large saccadic (motor) error (+1° to +2° and −1° to −2°) would falsely be detected as target jumps. With predictive remapping a false interpretation of the saccadic error as target displacement is less likely. In other words, Figure 9 illustrates how the SDD effect is a by-product of visual stability: Visual stability can be understood as perceiving the world as being displaced peri-saccadically only if it actually is displaced. Saccadic scatter leads to different post-saccadic retinal positions of the saccade target in each trial. Without the SSD effect, these different retinal positions would be misperceived as peri-saccadic displacements. Thus, SSD helps to avoid such misperceptions by increasing the threshold for the detection of peri-saccadic target displacements. Predictive remapping is involved in this increase of the threshold for displacement detection since it keeps the veridical pre-saccadic stimulus position in peri-saccadic “memory” thereby distorting new (displaced) sensory signals toward the memorized (veridical) position representation.

## 4. Discussion

Since its first discovery by Duhamel et al. (1992) there have been numerous reports of peri-saccadic dynamic RF updating. Some of these RF shifts might not exactly remap to the FRF location and rather shift into the direction of the focus of attention at the saccade target which leads for most previously tested spatial layouts to similar RF shifts as those of predictive remapping (Zirnsak et al., 2010; Hamker et al., 2011; Zirnsak et al., 2011b). However, in this study we investigate the potential role of the classical definition of predictive remapping which posits that a neuron starts responding to a stimulus presented in its FRF just before the eye movement (Duhamel et al., 1992). From early on, predictive remapping has been proposed as the central neural mechanism for the subjective experience of visual stability (Duhamel et al., 1992), an idea that has been further refined in recent reviews (Melcher and Colby, 2008; Wurtz, 2008), but neither its computational mechanisms nor its exact role in maintaining visual stability have been well understood (Hamker et al., 2011). We have focused on area LIP as one particularly important area for predictive remapping but similar mechanisms might also take place in the FEF (Sommer and Wurtz, 2006; Shin and Sommer, 2012).

While there is no widely accepted explanation about the underlying neural substrate of predictive remapping, a few computational models have explained predictive remapping in different ways. Quaia et al. (1998) explain remapping in LIP by means of a routing circuit. LIP cells are required to be all-to-all connected and the oculomotor-related signal from the FEF that indicates the saccade amplitude gates a particular set of connections to establish a predictive response. However, such all-to-all connection is biologically very unlikely. Keith et al. (2010) designed a variant of the Zipser and Andersen (1988) model to learn spatial updating in the double-step task. Interestingly, some cells in the model developed receptive fields that shift along the saccade vector. However, a reverse shift in other cells has also been observed. While the ability to learn receptive fields that remap is a great advantage of this model, learning is implemented in a supervised regime, that is, it requires a teacher signal about the correct output, which is unlikely to exist in LIP. Schneegans and Schöner (2012) recently proposed a model of remapping based on gain-fields which do not require all-to-all connections. However, remapping has been modeled using a gaze input with dual peaks in eye position, the present and future one, without further specifying where such eye position signals would originate. Our model differs from the one of Quaia et al. (1998) in that it does not require the massive all-to-all connections as it relies on the concept of gain fields. From the viewpoint of connectivity, our model only requires local (Gaussian) weight connections from the input/output maps to each neuron in the *Xb*_CD_ map where remapping takes place. Remapping in our model is an inherent aspect of coordinate transformation, but part of the feedback stream. While the feedforward streams do either code stimulus position with respect to present or future eye position, the feedback component encodes, given the predicted future eye position, the predicted stimulus location that would lead to the same head-centered response as the present stimulus position with respect to present eye position. Thus, remapping plays in our model a particular role in trans-saccadic perception where eye position information is used to align the pre-saccadic with the post-saccadic view and remapping anticipates the post-saccadic view.

Previous models of remapping mainly addressed the spatial updating of saccade targets (Quaia et al., 1998; Xing and Andersen, 2000; Keith and Crawford, 2008) or full field coordinate transformation (Schneegans and Schöner, 2012), but not its function in visual stability (Hamker et al., 2011). In this study we offer a comprehensive explanation of the function of predictive remapping in SSD. SSD has been previously explained by an object reference theory (Deubel et al., 1996; Bridgeman, 2007) and by an optimal transsaccadic inference theory (Niemeier et al., 2003). The object reference theory does not refer to predictive remapping and rather explains SSD as a build-in assumption of the brain, which aligns the pre-saccadic to the post-saccadic view according to available references, such as the saccade target. Niemeier et al. (2003) explain SSD on a rather abstract computational level. According to their Bayesian model, SSD represents optimal integration of all available information including a prior that transsaccadic position changes are unlikely events. To explain the blanking effect, however, they have to assume that this prior changes. While the conceptual framework of such transsaccadic integration is very interesting and relates to our model, such a Bayesian account does neither allow to specify the neurophysiological mechanism nor does it allow to make inferences about the temporal dynamics of perception around eye movements. Our physiology-based model adds to this rather abstract model in that it allows explaining the blanking condition by means of a dynamic circuit without any changes of parameters: Under normal transsaccadic conditions stimuli are available before and after saccade so that the pre-saccadic stimulation remains encoded in the dynamic neural network and affects the perception of the immediate post-saccadic stimulus. Blanking means that the appearance of the post-saccadic stimulus is delayed and thus according to our model, it occurs at a time where the trace of the pre-saccadic stimulus already decayed, so that now perception is not affected by the pre-saccadic view. Thus, our model predicts that blanking emerges from the temporal systems dynamics.

The potentially most important progress of our study does not necessarily lie in the novel explanation of a single particular experiment but in its ability to generalize across different studies. As the same model, using identical parameters, has been previously demonstrated to simulate the systematic localization errors of briefly flashed bars in complete darkness (Ziesche and Hamker, 2011), we show that SSD and the mislocalization of flashed stimuli in total darkness can be explained on an identical neural substrate involving remapping. How do then SSD and the mislocalization of flashed stimuli in total darkness relate to each other, why is such a system, that leads to perceptual errors, useful at all, and why can a single model based on predictive remapping account for these seemingly different experimental data? In experiments using flashed stimuli the visual system is probed in a brief period in time around saccade. Due to the corollary discharge signal, LIP(CD) cells start representing the stimulus with respect to a reference that already codes the future eye position (Figure 3) and thus, integrated over LIP(PC) and LIP(CD) cells, the stimulus is seen at a non-veridical position in direction to saccade. As an emergent result of the model's neural dynamics, the amount of mislocalization deceases with increasing flash location, which likely explains large variations in the amount of mislocalization across different studies (Dassonville et al., 1992; Van Wetter and Van Opstal, 2008; Van Grootel et al., 2012). In SSD the situation is different. Here the stimulus is visible already prior to the eye movement and its elicited neural activation inherently stabilizes any slightly displaced new population response, that enters the visual system after saccade, toward the pre-saccadic stimulus. Again, the LIP(CD) cells are crucially involved as the stabilization takes place within the circuit of LIP(CD) and intermediate cells. While SSD might appear as a shortcoming of the visual system, this suppression of displacement also compensates for inaccuracies in the saccade execution (saccadic scatter) (Kapoula and Robinson, 1986). Since such errors of the motor system can only be known by the visual system once post-saccadic proprioception about eye position is veridical, which can take longer than 100 ms after saccade offset (Xu et al., 2012), the visual system should have an increased threshold for transsaccadic stimulus displacements to avoid false alarms. Indeed, in our model simulations, the amount of suppression for peri-saccadic target displacements, which is produced by the stabilizing effect of the predictive remapping response, is sufficient to avoid false alarms to the displacements stemming from saccadic scatter (Figure 9). Thus, a necessary requirement for a stable perception of the visual world across eye movements is that no displacements of the world are detected as long as the world actually remains stable during the eye movements. Using our computational framework, we show that a predictive remapping response, which is generated by a corollary discharge of the saccade plan, prevents such false alarms of stimulus displacements and thus serves a stable perception of the visual world.

In sum, our model offers a fresh, new view onto the role of predictive remapping in the subjective perception of a stable world and provides several testable predictions. For example, it proposes the existence of two types of LIP cells as far as the extraretinal modulatory signal is concerned that can be either eye position or eye displacement, while mixtures are also possible. Second, it proposes that predictive remapping relies on an intact projection from eye position modulated to eye displacement modulated cells which is mediated by corollary discharge. The disruption of these connections, e.g., by suppressing corollary discharge should diminish predictive remapping. Third, any disruption of predictive remapping or corollary discharge should lead to a systematic bias in SSD opposite to saccade direction. Fourth, as the difference between the normal and the blanking SSD is explained by the modulatory effect of the pre-saccadic stimulus trace, any disruption of this trace should decrease the displacement threshold in SSD. Of course, a full account of perceptual stability requires testing how well the proposed mechanisms work when scaled up to more complex scenes. While interesting, the SSD paradigm is limited in that only a single stimulus is present and it might be that other mechanisms, such as the relative localization to visual landmarks (Bays and Husain, 2007), may be important in real world scenes. Similarly, the role of sustained visual attention has to be further explored (Golomb et al., 2010; Rolfs et al., 2011). However, our computational study provides a fundamental step toward an understanding of the role of predictive remapping since its first discovery by Duhamel et al. (1992). The model suggests that predictive remapping stabilizes the visual world across saccades by introducing a feedback loop. The stabilizing effect helps in avoiding misperceptions which can arise from motor errors in the saccade execution.

### Conflict of interest statement

The authors declare that the research was conducted in the absence of any commercial or financial relationships that could be construed as a potential conflict of interest.

## Supplementary material

Movie S1 **Simulation results of the model dynamics in a predictive remapping experiment as illustrated in Figure 3**. The top left panel shows the visual setup. The cross represents the center of gaze, the dot represents the stimulus position. The simulation shows a 20° saccade from the position of 10° toward the constantly shown stimulus at −10°. The top right panel shows the retinal input signal in the model layer Xr (on. both axes) After the stimulus latency of 50 ms, neurons in this layer start to respond to the stimulus. At time index 0 the eyes start to move (see top left panel). After the stimulus latency of 50 ms, Xr shows the gaze shift-induced movement of the stimulus response. The bottom left and bottom right panels represent the two simulated LIP populations *Xb*_PC_ and *Xb*_CD_, respectively. The retinal input from Xr enters both maps on the vertical axis. In *Xb*_PC_ (bottom left panel) the visual activity is modulated by the proprioceptive eye position signal which enters the map on the horizontal axis. Thus, before saccade the response in *Xb*_PC_ peaks at the pre-saccadic eye position of 10°. Peri-saccadically, the activity in this map is suppressed. Post-saccadically around 110 ms after saccade onset, suppression ends in *Xb*_PC_ and the activity updates to represent the post-saccadic eye position of −10°. In *Xb*_CD_ (bottom right panel) the visual activity is modulated by the corollary discharge signal, which enters the map on the horizontal axis at the future eye position (here −10°). In contrast to *Xb*_PC_, cells in *Xb*_CD_ respond to visual input even in the absence of the CD signal. The green circle indicate the cells which, according the classical formulation of predictive remapping, should show a predictive remapping response to the stimulus. Before saccade onset, the visual response in *Xb*_CD_ starts to show the effects of the rising CD signal. A remapping response is triggered by the CD signal which is centered at −10° and interacts with the input from the intermediate cells that enters *Xb*_CD_ diagonally along the dashed line. Thus, both signals together provide an additional input to the cells located within the green circle, exactly at the intersection of the diagonal dashed line with the center of the CD signal at −10°, just prior to saccade onset. This is the classical predictive remapping response.
